# ddRADseq reveals determinants for temperature-dependent sex reversal in Nile tilapia on LG23

**DOI:** 10.1186/s12864-017-3930-0

**Published:** 2017-07-14

**Authors:** Stephan Wessels, Ina Krause, Claudia Floren, Ekkehard Schütz, Jule Beck, Christoph Knorr

**Affiliations:** 1 Department of Animal Sciences, Division of Aquaculture and Water Ecology, University of Goettingen, Albrecht-Thaer-Weg 3, 37075 Goettingen, Germany; 2 Department of Animal Sciences, Division of Livestock Biotechnology and Reproduction, University of Goettingen, Burckhardtweg 2, 37077 Goettingen, Germany; 3 Institute of Veterinary Medicine, University of Goettingen, 37077 Goettingen, Germany; 4 Chronix Biomedical GmbH, 37077 Goettingen, Germany

**Keywords:** ddRADseq, F_ST_, Temperature, Sex reversal, LG23, Nile tilapia, Sex chromosomes

## Abstract

**Background:**

In Nile tilapia sex determination is governed by a male heterogametic system XX/XY either on LG1 or LG23. The latter carries a Y-specific duplicate of the *amh* gene, which is a testis-determining factor. Allelic variants in the *amh* gene demonstrated to be major triggers for autosomal and temperature-dependent sex reversal. Further, QTL on LG23 and LG20 show a temperature-responsiveness with influence on the phenotypic sex relative to the sex chromosomes. Here we present a ddRADseq based approach to identify genomic regions that show unusual large differentiation in terms of fixation index (F_ST_) between temperature-treated pseudomales and non-masculinized females using a comparative genome-scan. Genome-wide associations were identified for the temperature-dependent sex using a genetically all-female population devoid of *amh-ΔY*.

**Results:**

Twenty-two thousand three hundred ninety-two SNPs were interrogated for the comparison of temperature-treated pseudomales and females, which revealed the largest differentiation on LG23. Outlier F_ST_-values (0.35–0.44) were determined for six SNPs in the genomic interval (9,190,077–11,065,693) harbouring the *amh* gene (9,602,693–9,605,808), exceeding the genome-wide low F_ST_ of 0.013. Association analysis with a set of 9104 selected SNPs confirmed that the same genomic region on LG23 exerts a significant effect on the temperature-dependent sex.

**Conclusions:**

This study highlights the role of LG23 in sex determination, harbouring major determinants for temperature-dependent sex reversal in Nile tilapia. Furthermore F_ST_ outlier detection proves a powerful tool for detection of sex-determining regions in fish genomes.

**Electronic supplementary material:**

The online version of this article (doi:10.1186/s12864-017-3930-0) contains supplementary material, which is available to authorized users.

## Background

Genotyping-by-sequencing is a cost-effective approach to interrogate a multiplicity of loci simultaneously in large numbers of individuals [1]. In fact, only a fraction of homologous regions in the genome is sequenced and genotyped for available Single Nucleotide Polymorphisms (SNPs). Such approaches have proven successful in non-model organisms to identify genomic regions that determine complex traits such as the sex-determining loci in some bony fish [2–6]. Fish display a series of sex-determining mechanisms. Commonly, genetic sex determination (GSD) is divided into male heterogametic XX/XY or female heterogametic ZZ/ZW systems [7]. Moreover, pure environmental sex determination (ESD) exists, as well as transitions between the latter and GSD [7, 8]. Thus, chromosomal sex can be overlaid by extreme environmental conditions, such as elevated temperatures (reviewed in [8–10]).

Nile tilapia is a perciform fish from the cichlid clade, native to the Middle East and Africa. On the one hand, it alleviates hunger and constitutes an export commodity in light of its ability to thrive under poor conditions and ease of culture [11]. On the other hand, it is an excellent model for the intriguing complexity of sex determination and early stage sex chromosome development. Sex determination in Nile tilapia is orchestrated by major and minor genetic effects in addition to temperature [10]. Recent advances in short-read sequencing technologies and reduced representation genotyping approaches, have enabled the identification of sex-determining regions on linkage groups LG1 and LG23 in different Tilapia populations [3, 12–15]. RADseq revealed a major QTL for sex determination on LG 1 [3]. Moreover, whole genome sequencing of DNA pools from male and female tilapia detected a strongly differentiated inversion of 8.8 Mbp width, surrounding the putative sex-determining factor on LG1 [14]. This candidate region might be identically to the recently characterized *zfand3* gene. Though mapped in a hybrid of Mozambique tilapia and red tilapia, it showed a strict association between the phenotypic and genetic sex in the studied population [16]. The sex-determining factor on the sex chromosome LG23 is in fact a male specific duplication of the *amh* gene (*amh*y) [13]. Moreover, sex-skewing and temperature-dependent QTL reside on LG20, and LG23 [12, 13, 17, 18]. Allelic variants in the *amh* gene can lead to autosomal and temperature-dependent sex reversal [17].

The present study deals with a ddRADseq based methodology aimed at identifying regions of the genome with unusual large fixation index (F_ST_). Temperature-treated pseudomales and females, lacking the LG23 sex-determining *amhΔY,* were compared in a genome-scan. Furthermore, a genome-wide association study for the temperature-induced pseudomale phenotype was pursued using an *amhΔY*-negative genetically all-female population.

## Results

### Phenotypes and sex ratios

**Fig. 1:**
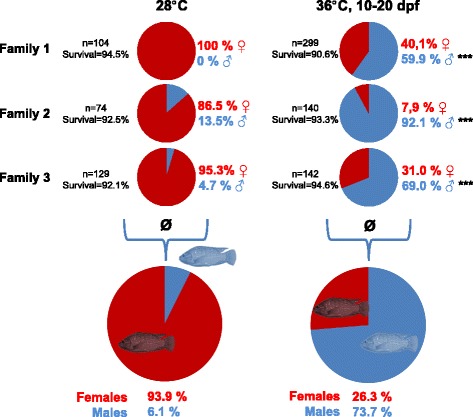
Sex ratios, numbers of sexed individuals, and survival from 10 dpf until sexing in control groups (28 °C) and temperature-treatment groups (36 °C, 10–20 dpf) of a genetically female population. Graphics were produced by the author. Copyright permissions were not required for the use of these graphics

Three genetically female (XX) families and one genetically supermale (YY) family were raised. Supermales were used in order to verify the presence of *amhΔY* as the putative sex chromosome in the investigated population. As the present study explicitly aimed to further decipher determinants for temperature-dependent sex reversal, exclusion of autosomal genes or other sex-skewing modifiers was paramount. As only family 1 was devoid of males in the control group, initially this family was chosen for the ddRADseq approach (Fig. 1). Subsequently families 2 and 3, which showed some sex-reversal in the control groups reared at 28 °C, too, were additionally sequenced using ddRADseq for a case-control approach. The genetically female full sibs were divided into a control (28 °C) and a treatment group (36 °C, 10–20 days post fertilisation (dpf)). The average male percentage of all control groups was 6.1% with sex ratios of 0%, 13.5% and 4.7% in families 1, 2, and 3, respectively. The overall male percentage in the three corresponding temperature-treated groups was 73.7% and differed significantly from the ratio in the controls (*p* < 0.0001). Despite that, each family showed significant deviations of the male proportion compared to the control groups (*p* < 0.0001), there were large differences in the temperature-responsiveness among families. Family 1 showed a male proportion of 59.9% in the temperature-treatment group, whereas families 2 and 3 beared 92.1% and 69.0% of males, respectively.

The population of YY-supermales contained no female offspring. Males were identified upon the presence of sperm. Further, some YY-supermales progeny were used as sires and were bred to normal females, resulting exclusively in all-male broods (data not shown).

### Survey of proposed sex determination loci *Oni23063*, *Oni28137*, and *amhΔY*

Comparative sequencing of the putative LG1 sex determination loci *Oni23063* and *Oni28137* [3] in sires and dams of the genetically all-female population revealed genotype G/G for SNP *Oni23063* and T/T for SNP *Oni28137*, respectively. The YY-individuals showed genotypes G/G at locus *Oni23063*, and T/T at locus *Oni28137*.

Furthermore, the genetic sex on LG23 was determined according to [19]. The 1252 bp PCR-fragment flanking exon 6 in *amh* was amplified in the 120 genetically female individuals and the 10 YY supermales. All YY supermales showed the expected cleavage pattern of *amhΔY*, resulting from the 5 bp insertion. Restriction analyses of the 120 genetically female individuals indicated no cleavage pattern, indicating that all individuals were devoid of the Y-specific *amhΔY* (Additional file 1: Fig. S1).

### ddRAD-tags and genome mapping

In total three genetically female families with 20 males and 20 females each were sampled and sequenced (Additional file 2: Table S1). All DNA samples were individually barcoded and indexed to be subsequently sequenced in 4 lanes NextSeq 500 and 3 lanes HiSeq 2000. Eighty-eight percent (175,697,219 reads) of the initial 200,643,953 single end reads were mapped to the Tilapia genome version Orenil1.1 (http://www.ncbi.nlm.nih.gov/assembly/GCF_000188235.2). Subsequent SNP calling, genotyping of the individuals and population genomic statistical analysis was performed using the ref_map.pl script of the Stacks program Version 1.34 [20]. Stacks first groups the aligned ddRADseq reads into genomic loci based on their position on the reference genome. Then polymorphic sites in each individual are identified and the most likely genotypes are inferred by applying a bounded-error statistical model, which accounts for sequencing errors. The *populations* script was applied in order to calculate population genomic parameters, such as pairwise F_ST_, kernel-smoothed F_ST_, expected and observed heterozygosity (H) as well as nucleotide diversity (π). Independent but equally treated runs of the *populations* script were performed comprising the following data sets: 1) comparison of 20 temperature-treated males with 20 females in family 1; 2) comparison of 60 temperature-treated males (affected cases) and 60 temperature-treated but non-masculinized females (unaffected controls) from families 1, 2, and 3. The ddRADseq analysis led to the discovery of 22,392 shared SNPs among temperature-treated pseudomales and females in family 1 (data set 1). A total of 9104 SNPs (data set 2) were detected amongst the genetically affected cases/unaffected controls and 5716 SNPs were shared between the data set 1 and 2.

### Genome-wide estimates of population differentiation between temperature-treated pseudomales and females

**Fig. 2:**
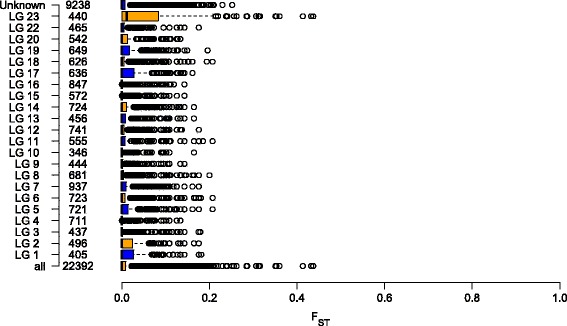
Boxplot of the genome- and chromosome-wide F_ST_ between temperature-induced males (XX) and non-temperature-reversed genetic females (XX). *Centre lines* show the medians; *box limits* indicate the 25th and 75th percentiles as determined by R software; whiskers extend 1.5 times the interquartile range from the 25th and 75th percentiles, outliers are represented by *dots*; Numbers right to y-axis correspond to the number of interrogated SNPs

**Fig. 3:**
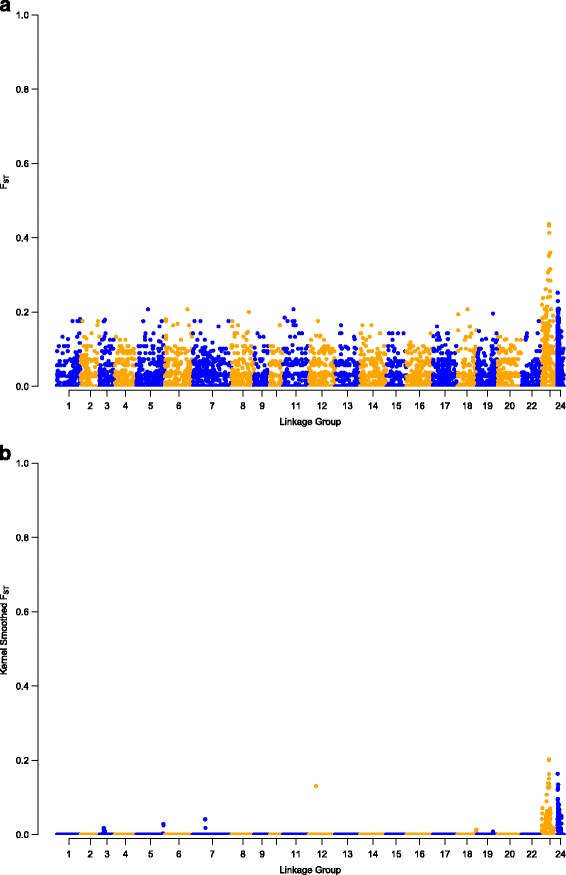
Genome-wide F_ST_ (**a**) and kernel-smoothed F_ST_ (**b**) between temperature-induced pseudomales (XX) and non-masculinized genetic females (XX)

**Fig. 4:**
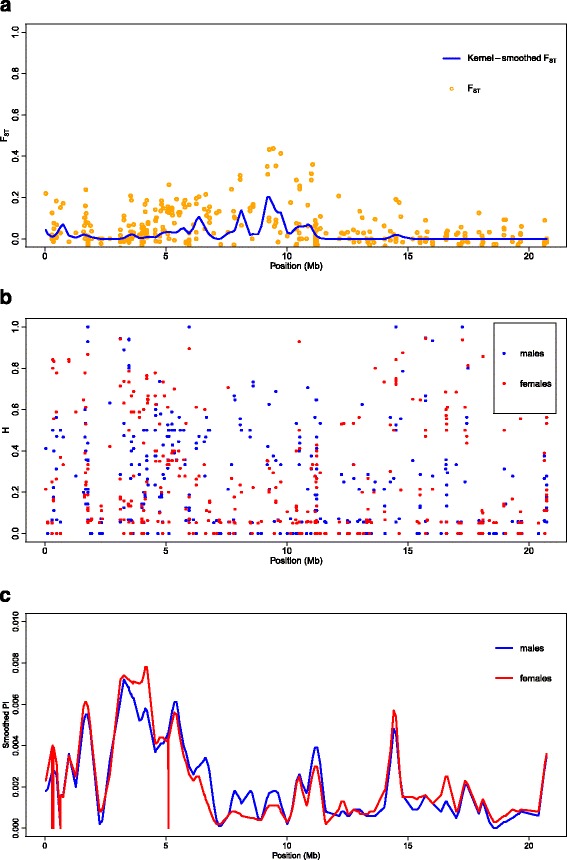
F_ST_ as well as kernel-smoothed F_ST_ (**a**), observed heterozygosity *H* (**b**), and kernel-smoothed nucleotide diversity *pi* (**c**) on LG23 between temperature-induced pseudomales and non-masculinized genetic females

**Fig. 5:**
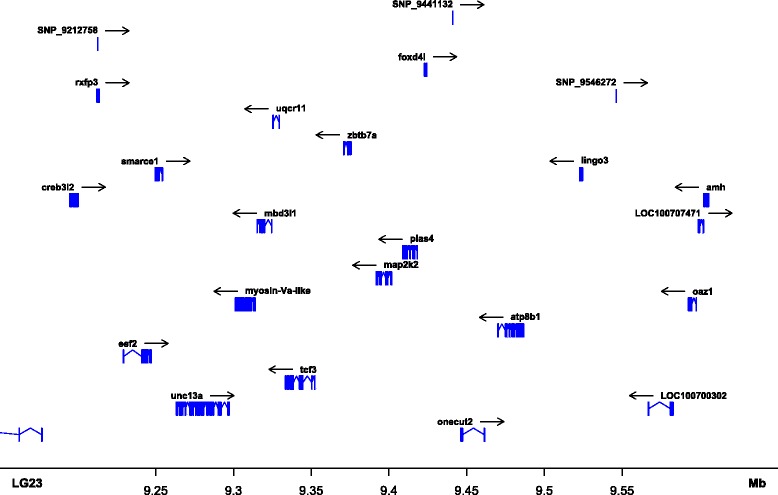
Detailed view of the region of largest differentiation between temperature-induced pseudomales and temperature-treated but non-masculinized genetic females on LG23. The region from 9,150,000 to 9,650,000, harbours 20 genes and 3 most significant SNPs. *Amh* is located at position 9,602,693–9,605,808

Using the obtained SNP genotype data, genome-wide differentiation between temperature-induced pseudomales and females for data set 1 was calculated. Average F_ST_ values of 0.0132 ± 0.0303 were obtained. Among the 22 linkage groups and unplaced scaffolds, LG23 showed the largest extent of differentiation between temperature-treated pseudomales and non-masculinized females, with a mean F_ST_ of 0.0519 ± 0.0787. Moreover, genome-wide extreme outlier loci exceeding the 99.9% quantile were observed exclusively on LG23 (Fig. 2, Additional file 3: Table S2). The most striking region on LG23 consisted of six SNP loci showing F_ST_ values larger than 0.3506 (Fig. 3). This region precisely corresponds with a region of both, reduced diversity (π) and heterozygosity (H) (Fig. 4), suggesting factors such as reduced recombination, low mutation rates or positive selection act on this genomic region. Within the corresponding 1875 Kbp interval of the Nile tilapia reference genome, 86 genes are located. The most striking candidate gene, namely the *amh* gene (ENSONIG00000004781, NC_022220: bp 9,602,068..9606447) has been flanked by three SNPs giving the highest observed F_ST_ values of 0.4322 at bp 9,280,012, of 0.4370 at bp 9,441,132, and of 0.41322 at bp 9,746,803. *Amh* and its Y-linked orthologues *amhΔY* and *amhy* have been identified as the sex-determining factor in some Nile tilapia populations [19]. Moreover, SNPs in the X-linked *amh* are known to trigger autosomal and temperature-dependent sex reversal [17]. Therefore, we aimed to confirm a consistently elevated F_ST_ in this genomic region and to test the hypothesis that it results from directional selection. Therefore a kernel-smoothed moving average (default parameter 3 σ base pairs to each side of the window) implemented in the Stacks software was applied. The F_ST_ as well as the kernel-smoothed F_ST_ both consistently peaked in the genomic region around the *amh* gene on LG23 (Figs. 3 and 4). Two more SNPs showed signs of directional selection. One of which was located at position 9,280,012 of LG23 in intron 14 of the *protein unc-13 homolog A-like* gene (LOC100702202), and one of which in an intron of the *lingo3* (ENSONIG00000021358) gene. Figure 5 summarizes the genes residing in this genomic region (9,150,000..9,650,000), Additional file 4: Table S3 deals with all genes on LG23.

### Association analysis for the temperature-dependent sex using case-control approach

**Fig. 6:**
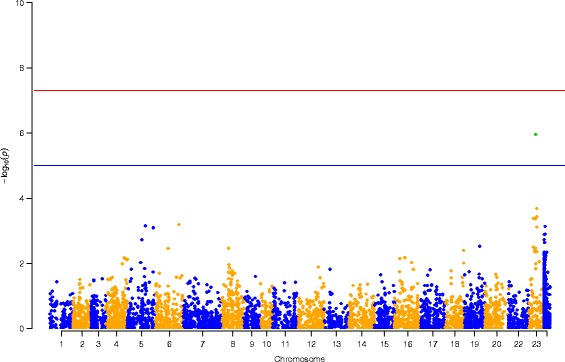
Manhattan plot of log-transformed *p*-values derived from basic association study between temperature-treated pseudomales (*n* = 60 affected cases) and non-masculinized genetic females (*n* = 60 unaffected controls). Asymptotic *p*-values were derived from basic allelic test chi-square with 1 degree of freedom (--assoc). All unplaced scaffolds are summarized as unknown (Unkn)

**Fig. 7:**
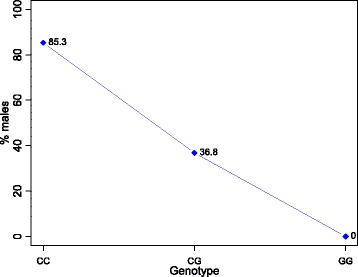
Relationship between SNP genotypes at position 9,441,132 on LG23 with temperature-dependent male proportion in Nile tilapia

In order to identify loci associated with the temperature-dependent sex, a case-control design was pursued using data set 2. In total, 5716 (22.2%) SNPs were intersecting with SNPs detected in data set 1. Association analysis using 9104 SNPs in data set 2 revealed that only 10 SNPs showed at least a suggestive association (*p* < 0.001; Table 1). Six of them were located on LG23 in the vicinity of the *amh* gene (Table 1). Interestingly, the SNP showing the highest association with the temperature-dependent sex was the closest to *amh* (Δ = 161 Kbp; Fig. 6). Moreover, suggestively significant SNPs resided on LG5 (*n* = 2), LG 6 (*n* = 1) and on scaffold 19 (GL831288.1; bp = 1,470,141). SNP 34055582 on LG5 is located in intron 2 of the *kif5a* gene. Gene ontology annotations for *kif5a* point to a role in ATPase and microtubule motor activity. SNP 23554897 is only 3 Kbp apart from the currently uncharacterized locus called 102,079,995. So far, none of these QTL had been linked to sex determination in Nile tilapia. Nevertheless, Bonferroni correction led to a reduction of significantly associated SNPs: only the four SNPs positioned on LG23 retained a significant association. After correction, all SNPs on LG23 flanking the *amh* gene showed a significant association with the temperature-dependent sex, with SNP 9,546,272, only 56 Kbp apart from *amh*. The strongest Bonferroni corrected association (*p* < 0.01) was observed for the SNP at position 9,441,132. At this locus the largest effect on the phenotypic sex was observed for genotype CC. 85.3% of all genotyped individuals were males (Fig. 7). Amongst heterozygotes 36.8% of the individuals were males. Most interestingly all homozygous G-allele carriers were females.

**Table 1:** Significant SNPs from case-control association analysis for temperature-dependent sex in Nile tilapia

LG	bp	N cases	N controls	Allele frequency in cases	Allele frequency in controls	Minor allele	Major allele	Chi-Square	P	Odds Ratio	L95	U95
5	23,554,897	43	46	0.163	0.391	A	G	11.49	0.000699	0.303	0.149	0.615
5	34,055,582	43	45	0.349	0.133	T	C	11.24	0.000800	3.482	1.641	7.389
6	30,817,177	42	51	0.012	0.157	C	T	11.66	0.000640	0.065	0.008	0.499
23	6,588,528	43	45	0.488	0.233	C	T	12.45	0.000419	3.136	1.644	5.984
23	9,212,758	53	53	0.170	0.387	G	A	12.42	0.000424	0.324	0.171	0.615
23	9,441,132	43	44	0.163	0.614	G	C	37.13	0.000001	0.122	0.060	0.250
23	9,546,272	45	46	0.267	0.609	A	G	21.6	0.003350	0.234	0.125	0.438
23	11,011,948	50	46	0.220	0.587	G	C	26.98	0.000206	0.199	0.106	0.372
23	11,011,949	49	47	0.235	0.596	C	T	25.83	0.000373	0.208	0.112	0.388
23	11,203,620	51	54	0.284	0.102	T	C	11.33	0.000764	3.503	1.642	7.472
24	1,470,141	44	55	0.193	0.418	C	G	11.41	0.000731	0.333	0.174	0.639

*LG* Linkage group, *bp* base pair, *cases* temperature-treated males, *controls* temperature-treated but non-masculinized females, *Chi-square* basic allelic test chi-square (1df), *P* asymptotic *p*-value for basic allelic test, *L95* Lower bound of 95% confidence interval for odds ratio, *U95* upper bound of 95% confidence interval for odds ratio

## Discussion

The sex determination in tilapia underlies a complex multi-stage mechanism. Effects of temperature on the phenotypic sex have amply been proposed [9, 17, 21–23]. Genomic approaches identified several linkage groups of the tilapia genome harbouring QTL and candidate genes for sex determination [18, 24–26]. The application of next generation sequencing approaches recently fostered the amount and quality of data for this trait [3, 12–14], but the question of the causative genetic background is still not answered. At least in some populations of Nile tilapia a male specific duplication of the *amh* gene (*amh*y) on LG23 constitutes the sex chromosome [13]. Moreover, besides major sex-determining factors on LG1, sex-influencing genes and genomic locations exist at least on LG20, and LG23 [12, 13, 17] that show a temperature-responsiveness with influence on sexual fate which can override the action of the major sex factor.

Previous studies demonstrated that SNP ss831884014 within the *amh*-gene (on LG23) is a major cue for both autosomal and temperature-dependent sex reversal. Here, we provide further evidence that LG23 and a 1875 Kbp interval genomic region encompassing the *amh* gene shows the largest evidence of differentiation between temperature-induced pseudomales and temperature-treated non-sensitive females. The present study revealed sex-specific patterns of genetic variability and pseudomale specific alleles at SNPs in close vicinity of the *amh* gene, whose male specific duplication (*amhy*) acts as a sex-determining factor on the LG23 sex chromosome [13]. Here, we also provide evidence for the presence of the *amhΔY* as the putative Y-chromosome in this Nile tilapia population from Lake Manzala, Egypt.

The investigated all-female population was devoid of *amhΔY,* whereas fish derived from a genetic supermale YY-line, but originating from the same population as the genetically female (XX) line, were all harbouring the y-specific *amhΔY.* Nevertheless, LG1 genotypes of the all-female population exhibited homozygous allelic states at locus *Oni23063* (G/G) as well as for SNP *Oni28137* (T/T), respectively. YY-individuals were homozygous for the G-allele at locus *Oni23063*, as well as the T-allele at SNP *Oni28137*. Palaiokostas et al. similarly determined the G-allele in homozygous state in genetic females at *Oni23063* and *Oni28137*, respectively [3]. Genetic males were heterozygous A/G and G/T at *Oni23063* and *Oni28137* [3]. Nevertheless, the presence of *amhΔY*, as wells as the GG-genotype at *Oni23063* in the investigated XX- and especially YY-individuals might indicate that the former acts as sex-determining factor, and that LG23 might represent the sex chromosome in this population. Under this hypothesis, the large allelic heterogeneity between temperature-treated pseudomales and females on LG23, and more specifically in the genomic region encompassing the *amh* gene, might likely indicate that on this early stage sex chromosome, the recombination rate plays a crucial role. Besides, in light of the confined genomic region in which we find evidence for loci influencing temperature-dependent sex reversal, sex-skewing minor loci, as well as the sex-determining factor *amhΔY* it is tempting to postulate that LG23, sex chromosomes and temperature-dependent sex factors are co-evolving in the same genomic region. Perrin et al. stated in fact that since recombination rather depends on the phenotypic than the genetic sex, a rapid decay of proto-sex chromosomes might occur, especially when sex reversed XY females are frequent [27]. Sex-specific recombination had been observed earlier in sex-reversed pseudomale tilapia, which showed male-specific synaptonemal complexes [28]. One possible mechanism explaining the high degree of differentiation between temperature-induced pseudomales and non-masculinized females in vicinity of the *amh* gene might be the phenotypically male-specific recombination pattern observed in this mating between a temperature-induced pseudomale and female. Thus, also haplotypes fostering temperature-dependent sex reversal could thus be conserved more easily. Consequently, if genetic females carrying allelic variants that nurture temperature-responsiveness would mate with genetic males (XY) with subsequent exposition of fry to elevated temperature, under the assumption that the same loci also lead to XY sex reversal, then recombination between the X and Y chromosome could take place. According to [27] this might lead to the breakdown of the evolving Y-specific haplotypes, which in turn would diminish due to the natural selection pressure. Thus, in light of a hampered Muller’s ratchet the homomorphism between X and Y persists. However, the data presented here is not sufficient to support this hypothesis entirely, but it should be one road of enquiry for future studies.

In Nile tilapia a rapid turnover of sex chromosomal systems has taken place. Depending on the strain of *O. niloticus* either a XY-system on LG1 or on LG23 dominates [14, 29]. Although a QTL for temperature-dependent sex was proposed for LG1 in an earlier work [18], we didn’t find evidence of elevated genetic differentiation between temperature-treated pseudomales and non-masculinized females, as were found on LG23. More precisely, even in the genomic region harbouring the putative causative sex-determining factor in some *O. niloticus* strains, contained by loci *Oni23063* and *Oni28137*, neither signs of genetic differentiation between temperature-induced pseudomales and non-masculinized females nor an association of loci with the temperature-dependent sex was found. However, sex-specific allelic patterns observed on LG23 might point to a rapid evolution of this young sex chromosome and possibly temperature-dependent sex reversal. Further, it confirms that LG23, the *amh* gene and modifications in the TGF-ß pathway play an integral role in evolution of sex-determining mechanisms in tilapia. Previously it was shown that allelic variants in the *amh* gene might lead to both temperature-dependent and spontaneous sex reversal, suggesting that at least partially the same genetic cascade is responsible, with more pronounced effects fostered by temperature treatment [17]. The LG23-wide elevated mean F_ST_ (Fig. 2) as well as the kernel-smoothed F_ST_ both consistently peaked in the genomic region around the *amh* gene indicating that it might in fact have been under directional selection (Fig. 4). Furthermore, another *TGF-ß* related gene *pias4* (ENSONIG00000004799) is located in this genomic region, which might, if LD persists, be subjected to directional selection, too. However, no markers were found within the *pias4* gene to prove this hypothesis. Moreover, another SNP, that showed signs of directional selection, was located at position 9,280,012 of LG 23 positioned in intron 14 of the *protein unc-13 homolog A-like* gene (LOC100702202). Furthermore, an intronic SNP in *lingo3* (ENSONIG00000021358) was identified that is also suspected to be under directional selection. Lingo3 is a transmembrane protein localized in the plasma membrane, however its role is yet unknown, too.

The loci identified here exert a large effect on the formation of the temperature-dependent sex; too, however the connection to spontaneous sex reversal remains to be elucidated. Palaiokostas et al. reported a sex QTL on LG20 which plays an ambiguous role during temperature-dependent as well as spontaneous sex reversal [12]. The here presented approach combining population genomics and association mapping failed to recover the proposed QTL on LG20, despite the fact that the genetically female population investigated here showed a high average male proportion (73.7%) in the temperature-treatment group. Moreover, although all families were genetically female (XX) and were derived from temperature-treated families whose untreated full sibs were also all-female, two thirds of the control groups exhibited some male individuals (Fig. 1). Genetic drift could be one reason for the spontaneous presence of those males in control groups. Alternatively, the fact that both parents had been temperature-treated might also have led to transgenerational epigenetic effects, leading to masculinization of individuals reared under the control temperature of 28 °C. A comparative analysis of spontaneously sex-reversed males and genetic females in controls is further needed in order to shed more light on the intermingling nature of sex chromosomes, loci associated to temperature-dependent sex, as well as loci promoting spontaneous sex reversal.

## Conclusions

The comparative genome-scan of temperature-treated pseudomales and non-masculinized females provides evidence that temperature-dependent sex reversal is evolving on a putative sex chromosome in Nile tilapia. Moreover, the present study indicates through the large extent of allelic variation in the genomic region harbouring the putative sex-determining gene *amh*/*amhy*, that LG23 might still be considered a very young and fairly homomorphic sex chromosome. Further research is needed in order to investigate the role of recombination and recombination suppression respective of the phenotypic sex, as well as to understand the co-evolution of sex chromosomes and temperature-dependent sex reversal. Additionally, research is needed to clarify the role of epigenetics and the life-history memory, as well as expression of gene networks during temperature-dependent sex reversal.

## Methods

### Animals and tissue collection

Breeding and rearing of genetically female XX and all-male YY Nile tilapia (*Oreochromis niloticus*, Lake Manzala, Egypt) was carried out at the warm water recirculation unit of the Division of Aquaculture and Water Ecology at the Department of Animal Sciences at the University of Goettingen. To obtain genetically all-female (XX) progenies, eggs were derived from temperature-treated F1-females from a cross between a weakly temperature-responsive (low-line) and strongly temperature-responsive (high-line) selection line [23, 30]. To obtain the selection lines, family selection was carried out upon the male percentages in treatment groups [23]. After three generations of divergent selection 50.4% males were observed after 36 °C treatment from 10 to 20 dpf in the low-line, whereas in the high-line 92.7% males were present [30]. Eggs from temperature-treated F1-females were fertilised with sperm from one of three high-line temperature-induced F1-pseudomales and incubated at 28 °C. Hatching occurred at ~4 dpf. After yolk sac absorption, larvae from each of the three families were randomly distributed into control and treatment groups (n ~ 110 larvae per tank, if available more fry were reared in separate tanks). Temperature in the control groups was permanently kept at 28 °C, whereas treatment groups were reared at an elevated temperature of 36 ± 0.5 °C from 10 to 20 dpf. Control and treatment groups were reared in 2 l plastic aquaria. Fry were fed ad libitum and were reared family-wise under a photoperiod of 12 h light and 12 h darkness. From 20 dpf onwards, the fish from control and treatment groups were raised at 28 °C in separate 80 l tanks for at least 3 months. Thereafter, fish from groups treated at high temperature and control groups were subjected to anaesthesia using essential oil of cloves (at 0.05 mL/L) and were immediately exanguated, dissected, and a fin clip was sampled and stored at −20 °C until DNA extraction. The sex of all fish was assessed based on microscopical inspection of squashed gonads according to [31], classifying them into either testes or ovaries.

For the production of YY-supermales, eggs were derived from a hormone-induced YY-pseudofemale and sperm was stripped from a single YY-male originating from a line developed by [32]. All YY-individuals were solely reared at 28 °C.

All procedures were in strict accordance with the recommendations in the Guide for the Care and Use of Laboratory Animals of the German Animal Welfare Act [33].

### DNA extraction

Genomic DNA was extracted from fin-clips using the DNeasy blood and tissue kit according to the manufacturer’s protocol (Qiagen, Hilden, Germany). All DNA samples were RNase-treated according to the manufacturer’s recommendations.

### Genotyping sex determination regions on LG1 (*Oni23063 and Oni28137*) and LG23 (*amh/amhΔY)*

Flanking sequences to amplify SNP *Oni23063* and *Oni28137* were derived from Scaffold NC022199.1. The genomic sequence of the *amh* gene was derived from Scaffold GL831234.1 of the Nile tilapia genome sequence deposited in the Ensembl database (http://www.ensembl.org; Orenil1.0 GCA_000188235.1; location of *amh*: Scaffold GL831234.1, 1.688.687–1.691.779). Gene- and locus-specific primers were designed using the Primer3 software (see Additional file 5: Table S4). Primers were used to amplify a specific fragment of 561 bp harbouring SNP *Oni23063* and *Oni28137* as well as another 1252 bp long PCR fragment flanking exon 6 of *amh*. PCRs were carried out using 20 ng of genomic DNA, 1× PCR buffer containing MgCl_2_, 1× Q-solution, 10 pM of each primer, 10 mM dNTPs and 2 U FastStart Taq DNA polymerase in a final volume of 25 μl. All PCR components, except for primers (MWG-Biotech, Ebersberg, Germany) and the 1× Q-solution (Qiagen, Hilden, Germany) were purchased from Roche (Roche,Penzberg, Germany). PCR was performed using a Biometra T-3000 Thermocycler (Biometra,Goettingen, Germany) with an initial denaturation at 95 °C for 10 min, followed by 35 cycles of 92 °C for 30 s, 60 °C for 30s and 72 °C for 1 min with a final extension at 72 °C for 5 min. The fragment identity was controlled via gel-electrophoresis on 1.5% agarose gels. Primers for *amh* were specific for all three reported forms of *amh*, i.e. the X-linked *amh*, and the Y-specific *amhy* homolog as well as *amhΔY* [19]. First of all *amh* was screened for 120 genetically female individuals and the 10 YY supermales. *AmhΔY* features a 5 bp insertion in Exon VI (ATGTC), which contains a *Taq* ^α^I specific recognition site. Therefore, all amplicons were *Taq* ^α^ *I* (NEB, Frankfurt, Germany) digested and separated on 2% agarose gels. The X-linked *amh* and the Y-linked *amhY* were represented by the amplicon of 1252 bp length, whereas, the Y-linked *amhΔY* was represented by cleaved fragments of 829 and 423 bp in length. In addition to the restriction digest, all results were further confirmed using Sanger sequencing. M13-tailed primers enabled direct bidirectional sequencing on an ABI-PRISM 3100® capillary analyser (Life Technologies, Darmstadt, Germany) using the Big Dye terminator Kit. PCR products were before purified with Exo-SAP-IT® (Thermo Fisher Scientific, Schwerte, Germany). The obtained sequences were trimmed, contigs were built, and SNPs were manually identified using the program software suite DNASTAR LasergeneTM6® (DNASTAR, Madison, WI, USA). In Nile tilapia populations with an LG1 sex chromosome, genetic females are supposed to carry genotypes G/G and T/T at loci *Oni23063* and *Oni28137*, respectively [3]. The available genome reference sequence of *O. niloticus* exhibits G/G at locus *Oni23063* and T/T at *Oni28137,* as it was derived from an individual of an XX homozygous isogenic line. Genetic males are supposed to be heterozygous showing genotypes A/G and G/T for loci *Oni23063* and *Oni28137*, respectively [3].

### ddRAD library preparation and sequencing

A modified approach of the ddRADseq protocol developed by [34] was pursued. DNA from each individual (500 ng) was digested using 0.5 μl *Eco*RI (20.000 U/ml) (specific for G|AATT|C recognition site) and 1 μl *Alu*I (10.000 Units/ml) (specific for AG|CT recognition site) in 1× NEBuffer 2.1 (NEB, Frankfurt, Germany) at a final volume of 30 μl. Digestions were run at 37 °C for 3 h and at a holding period at 4 °C without heat inactivation. Individual digests were then purified using a 2× volume of AMPure XP beads. Custom double-stranded sequencing adapters were designed for sticky end ligation to the digested DNA. An *Eco*RI compatible 5′-phosphorylated overhang was added to the original Illumina P5 adapter sequence and the *EcoR*I sequence was followed by an individual 5 bp barcode sequence, which was read at the beginning of each P5 sequencing read. One unique barcode was used for library preparation of each individual animal. The Illumina P7 adapters were designed to carry an *Alu*I compatible 5′-phosphorylated overhang and carried a biotin molecule (Additional file 2: Table S1). Both strands of each adapter were ordered as single stranded oligonucleotides (MWG-Biotech, Ebersberg, Germany) and were annealed in Ligase Buffer (NEB, Frankfurt, Germany) to form double-strands using the following temperature profile: 95 °C 1 min, 80 °C > 21 °C with 1°/sec, 21 °C 20 min. The resulting double-stranded adapters (375 nM each) were ligated to the digested DNA at room temperature for 30 min, using, 4 μL T4 DNA Ligase (USB/Affymetrix, Santa Clara, CA, USA) in 1× Ligase Buffer (NEB, Frankfort, Germany) and a total volume of 40 μL. After an additional purification with 2× volume of AMPure XP beads each sample was quantified using the Quant-it™ ds DNA Assay Kit (Thermo Fisher Scientific, Waltham, MA, USA) and a GENios Pro microplate reader (Tecan, Crailsheim, Germany). Samples were subsequently pooled in an equimolar way. The use of one biotinylated and one non-biotinylated adapter allowed the specific selection of proper P5-P7 library fragments by selective binding to streptavidin-coated beads and subsequent denaturing of the bound double-strands as described [35]. Binding and wash steps to 30 μL Dynabeads® MyOne (Thermo Fisher Scientific, Schwerte, Germany) were carried out according to manufacturer’s protocol. P5-P7 strands were eluted by denaturing the double-stranded DNA with alkaline lysis buffer: 400 mM KOH, 10 mM EDTA, 80 mM DTT. Each eluted library pool was diluted 1:10 and 2 μL were used as template in the final library PCR containing 100 μM of each dNTP, 2 units FastStart Taq DNA Polymerase, 1 μL 50xEvaGreen© and 400 μM P5-Universal-Primer and 400 μM P7-Index-Primer (Additional file 2: Table S1). Finally, 6 pools were size-selected to ~200–500 bp long fragments using an E-Gel electrophoresis system (Thermo Fisher Scientific, Waltham, MA, USA) and quantified using a 2100 Bioanalyzer (Agilent Technologies, Santa Clara, CA, USA). Libraries were then sequenced either on the Illumina HiSeq 2000 or the Illumina NextSeq 500 system, yielding reads with maximum single end read length of 100 or 151 bp, respectively (Additional file 6: Table S5).

### Raw read filtering, processing and population genomic analysis

The resulting raw single-end reads were demultiplexed using a custom Perl script allowing 1 mismatch in the adapter sequence. Reads shorter than 95 bp were filled with ‘N’. All reads were then truncated to 95 base pairs, quality filtered, and merged into one file per individual. Reads were subsequently mapped onto the Tilapia genome version Orenil1.1.

(http://www.ncbi.nlm.nih.gov/assembly/GCF_000188235.2) using the programme package BWA [36]. All resulting BAM-files were further analysed using the Stacks program Version 1.34 [20]. The datasets generated and analysed during the current study are available in the NCBI Bioproject repository, (Accession: PRJNA354565;https://www.ncbi.nlm.nih.gov/bioproject/?term=PRJNA354565). First, ddRAD tags were analysed using the *ref_map.pl* script, with a minimum stack depth of 5 (−m 5), allowing 1 mismatch between loci when building the catalogue (−n 1). The file *catalog.snps.tsv* was screened for erroneous SNP alleles resulting from in silico elongation of reads to 95 bp. All loci containing the erroneous ‘N’-allele were blacklisted using the –B option in subsequent analyses.

Secondly, the *population* script was applied in order to calculate population genetic parameters. As the present study explicitly aimed to further decipher determinants for temperature-dependent sex reversal, exclusion of autosomal genes or other sex-skewing modifiers was paramount. As only family 1 was devoid of males in the control group, initially this family was chosen for the ddRADseq approach. Subsequently families 2 and 3, which showed some sex-reversal in the controls, too, were additionally sequenced for a case-control approach. Independent runs of the *populations* script were performed comprising the following data sets: data set 1) comparison of 20 temperature-treated males with 20 females in family 1; 2) comparison of 60 temperature-treated males (affected cases) and 60 temperature-treated but non-masculinized females (unaffected controls) from families 1, 2, and 3. All data sets were filtered equally to a stack depth of >5 (−m 5), a minor allele frequency of >0.01 (--min_maf 0.01), and a minimum percentage of individuals in the population required to process the locus >70% (−r 0.7), ddRAD tags were requested to be present in all populations within a data set (−p 2). SNP and haplotype-based F-statistics were requested using the --fstats command, kernel smoothing of F_ST_ was enabled using the –k option applying a default window size of 3σ (150 Kbp). The fixation index F_ST_ was calculated with Stacks 1.34 [20] using an adapted formula, accounting for unequal sample size among populations by weighting [37]:

$$ {F}_{ST}=1-\frac{\sum_i\left(\begin{array}{c}\hfill {n}_j\hfill \\ {}\hfill 2\hfill \end{array}\right){\pi}_j}{\pi_{.}{\sum}_i\left(\begin{array}{c}\hfill {n}_j\hfill \\ {}\hfill 2\hfill \end{array}\right)} $$

where *n*_*j*_ is the number of alleles sampled in population *j*, *p*_*j*_ is the nucleotide diversity within population *j*, and *π* is the total nucleotide diversity across the pooled populations. Expected and observed heterozygosity (H) as well as nucleotide diversity (π) were calculated using the *populations* script.

### Association analysis

Temperature-dependent sex loci were identified in a case-control study (data set 2). Hence, 60 temperature-treated males (affected cases) and 60 temperature-treated but non-masculinized females (unaffected controls) of the above characterized three families were included. Genotypes of 9104 loci were obtained using Stacks program (version 1.34) [20] after running the *populations* script, applying the filters as used for data set 2. Association analysis of segregating SNPs was carried out using the software package PLINK [38]. Sex was coded as a binary trait with temperature-treated but non-masculinized females as 1 (unaffected/ control) and temperature-treated males as 2 (affected/ case). A case-control association analysis (--assoc) was undertaken. Subsequently a Bonferroni correction (--assoc --adjust) was applied during association analysis.

## Additional files

Additional file 1: Figure S1. *Amh* and *AmhΔY* genotypings for 60 temperature-treated pseudomales (affected cases) and 60 non-masculinized genetic females (unaffected controls) as well as 10 YY supermales. (PDF 252 kb)

Additional file 2: Table S1. Overview of P5-Universal- and P7-Index-Primer sequences for 60 temperature-induced pseudomales and non-masculinized females. (XLSX 17 kb)

Additional file 3: Table S2. Number of temperature-treated pseudomales (affected cases) and non-masculinized genetic females (unaffected controls) in the region of LG23 showing the largest F_ST_. (XLSX 29 kb)

Additional file 4: Table S3. Overview of refseq genes residing on LG23. (XLSX 234 kb)

Additional file 5: Table S4. Forward and reverse primers tailed with a universal M13 forward or reverse primer for bidirectional sequencing of the *amh* gene. Forward and reverse primers for sequencing LG1 loci *Oni23063* and *Oni28137*. (XLSX 11 kb)

Additional file 6: Table S5. Overview of number of reads, mapped reads as well as unmapped reads per individual. (XLS 46 kb)

## Abbreviations

bp: Base pair
ddRADseq: Double digest restriction site associated DNA sequencing
dNTP: Deoxynucleotide
dpf: Days post fertilisation
F_ST_: Fixation index
H: Heterozygosity
Kbp: Kilo base pairs
LD: Linkage disequilibrium
LG: Linkage group
Mbp: Mega base pairs
mM: Millimolar
ng: Nanogram
nM: Nanomolar
PCR: Polymerase chain reaction
pM: Picomolar
QTL: Quantitative trait loci
RADseq: Restriction site associated DNA sequencing
SNP: Single nucleotide polymorphism
TGF-ß: Transforming growth factor ß
U: Unit
μL: Microliter
μM: Micromolar
π: Nucleotide diversity
