# Supplementary figures and images for: ddRADseq reveals determinants for temperature-dependent sex reversal in Nile tilapia on LG23
Source: BMC Genomics. 2017 Jul 14;18:531. doi: 10.1186/s12864-017-3930-0 (PMC5513378; doi:10.1186/s12864-017-3930-0)

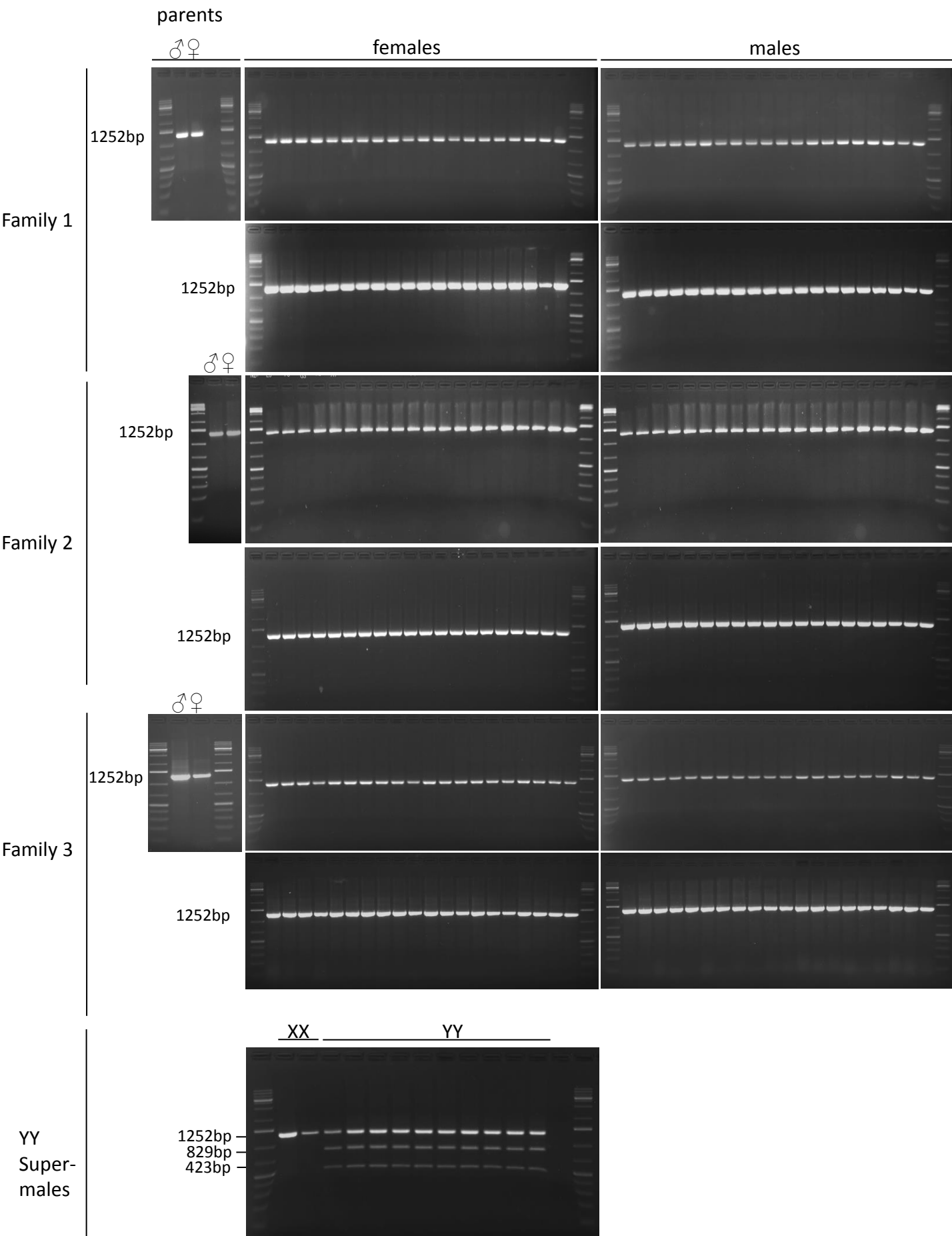

Supplement: Supplementary file 1 — Amh and AmhΔY genotypings for 60 temperature-treated pseudomales (affected cases) and 60 non-masculinized genetic females (unaffected controls) as well as 10 YY supermales. (PDF 252 kb) [file 12864_2017_3930_MOESM1_ESM.pdf]
